# Comparison of the Genedia MTB/NTM Detection Kit and Anyplex plus MTB/NTM Detection Kit for detection of *Mycobacterium tuberculosis* complex and nontuberculous mycobacteria in clinical specimens

**DOI:** 10.1002/jcla.23021

**Published:** 2019-09-16

**Authors:** Jihoon Kim, Qute Choi, Jong Wan Kim, Seon Young Kim, Hyun Jin Kim, Yumi Park, Gye Cheol Kwon, Sun Hoe Koo

**Affiliations:** ^1^ Department of Laboratory Medicine, Chungnam National University College of Medicine Chungnam National University Hospital Daejeon Korea; ^2^ Department of Laboratory Medicine, Dankook University College of Medicine Dankook University Hospital Cheonan Korea

**Keywords:** *Mycobacterium tuberculosis* complex, non‐respiratory specimen, nontuberculous mycobacteria species, respiratory specimen

## Abstract

**Background:**

Real‐time (RT) PCR is a rapid and accurate method that is widely used for the detection of *Mycobacterium tuberculosis* complex (MTB). The aim of this study was to evaluate and compare the performance of the Genedia MTB/NTM Detection Kit and the Anyplex plus MTB/NTM Detection kit in the detection of MTB and nontuberculous mycobacteria (NTM) in clinical specimens.

**Methods:**

From October 2017 to February 2018, 236 respiratory specimens and 137 non‐respiratory specimens, from patients with suspected tuberculosis, were examined. AFB smear, culture, and RT‐PCR using the Genedia MTB/NTM Detection kit (Green Cross Medical Science Corp.) and the Anyplex plus MTB/NTM Detection kit (Seegene) were applied. PCR performance in the detection of MTB and NTM was evaluated in relation to culture results and between the two assays.

**Results:**

Culture was positive for MTB in 30 (8.0%) of the 373 specimens and for NTM in 23 (6.2%). The sensitivity and specificity of MTB detection with the Genedia kit were 76.7% and 99.7%, respectively, whereas the Anyplex kit sensitivity and specificity for MTB detection were 86.7% and 97.5%, respectively. Both kits exhibited the same sensitivity (73.9%) for NTM detection, and the specificity was 100% and 99.4% for the Genedia and Anyplex kits, respectively.

**Conclusions:**

The Genedia and Anyplex kits demonstrated high sensitivity and specificity for the detection of MTB and NTM. Both kits have a high concordance rate and can be used more widely in clinical laboratories for the early detection of tuberculosis.

## 1 INTRODUCTION

Worldwide, tuberculosis (TB) is one of the top 10 causes of death and the leading cause from a single infectious disease. In 2017, there were an estimated 1.3 million deaths due to TB among HIV‐negative people and an additional 300 000 deaths among HIV‐positive people. About 6.4 million new cases of TB were officially notified to the World Health Organization (WHO) via national authorities, although TB incidence and mortality rates are gradually decreasing year after year.^1^

*Mycobacterium tuberculosis*, the causative agent of tuberculosis, can be detected using a variety of methods, such as acid‐fast bacillus (AFB) staining, culture, and PCR. Specifically, molecular methods, using PCR‐based techniques, can be used to obtain accurate results in a short period of time and are typically used in conjunction with conventional culture and microscopy‐based methods.^2^

The Genedia MTB/NTM Detection kit (Green Cross Medical Science Corp.) was developed for the rapid molecular detection of the *M tuberculosis* complex (MTB) and nontuberculous mycobacteria (NTM). The Genedia assay is a real‐time (RT) PCR method targeting IS*6110* with TaqMan hydrolysis probes for MTB and the rpoB gene for NTM. In this present study, we evaluated the performance of the Genedia kit using clinical respiratory specimens as well as non‐respiratory specimens. Then, we compared the results with the Anyplex plus MTB/NTM Detection kit (Seegene).

## 2 MATERIALS AND METHODS

### 2.1 Samples

We selected respiratory and non‐respiratory samples that were transferred to the Department of Laboratory Medicine in a tertiary university hospital for MTB/NTM PCR from October 2017 to February 2018. All clinical samples were examined by AFB smear, conventional culture, and RT‐PCR. This study was approved by the institutional review board of Chungnam National University Hospital.

### 2.2 AFB smear and culture

Respiratory specimens were treated with NALC‐NaOH (2% N‐acetyl‐L‐cysteine–sodium hydroxide) after centrifugation at 3000 × *g* for 20 minutes. The AFB smear procedure involved staining with auramine‐rhodamine fluorescent stain, followed by confirmation using Ziehl‐Neelsen staining. Smears were graded, and those from 1 to 4 were defined as smear‐positive. All specimens were cultured in liquid (MGIT960 system) and solid (2% Ogawa agar) media for a maximum of 6 and 8 weeks, respectively. Identification of MTB and NTM was carried out by MPT 64 antigen detection using immunochromatographic method.

### 2.3 Real‐time PCR kits

RT‐PCR was performed using two kits (Genedia MTB/NTM Detection kit and Anyplex plus MTB/NTM Detection kit) according to the manufacturers’ instructions. One hundred microliters of decontaminated sample was mixed with 100 µL DNA extraction solution from each assay kit and heated at 100°C for 10 and 20 minutes, for the Genedia and Anyplex assays, respectively. Each total mixture including PCR mixture, primer mixture, internal control DNA, and template DNA was centrifuged and prepared for PCR reactions according the manufacturers’ instructions. Assays were run on a CFX96TM real‐time PCR system (Bio‐Rad). Positive and negative controls were included in each run for both instruments. Results were automatically interpreted via the instrument software, using pre‐defined threshold and cutoff values.

### 2.4 Statistical analyses

Sensitivity, specificity, positive predictive value, and negative predictive value were organized using Microsoft Excel 2013 software (Microsoft). The kappa value was calculated using SPSS for Windows (IBM SPSS Statistics version 18.0).

## 3 RESULTS

**Figure 1:**
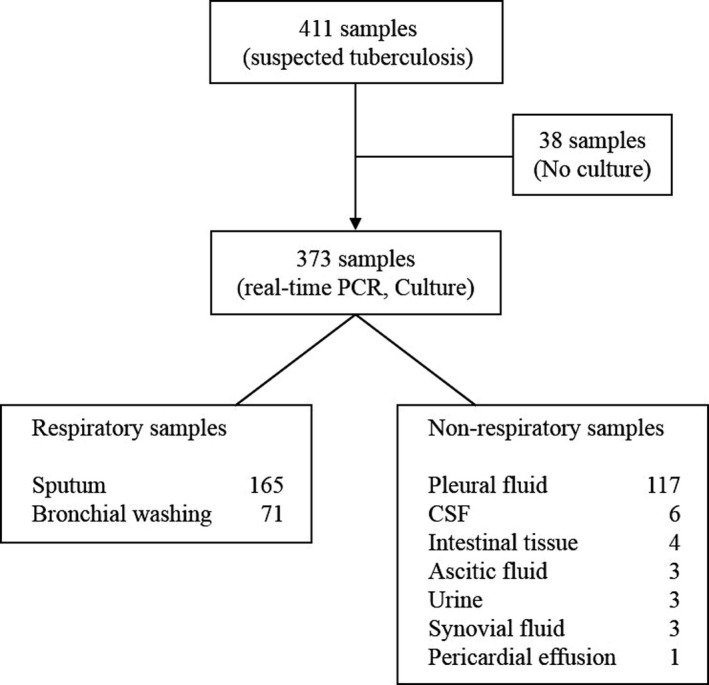
Distributions of tests performed for respiratory and non‐respiratory specimens. CSF, cerebrospinal fluid

Of the 411 clinical specimens collected, 38 were excluded due to missing culture results. The remaining 373 samples consisted of 236 respiratory specimens (165 sputa, 71 bronchial washings) and 137 non‐respiratory specimens (117 pleural fluid, 6 CSF, 4 intestinal tissue, 3 ascitic fluid, 3 urine, 3 synovial fluid, 1 pericardial effusion) (Figure 1).

Specimens were examined by AFB smear, culture, and using both RT‐PCR tests. Of these, 37 (9.9%) were AFB‐positive, including 36 respiratory specimens and 1 synovial fluid specimen; the remaining 337 (90.3%) specimens were AFB‐negative (Table 1).

**Table 1:** Performance of two real‐time PCR kits based on the AFB smear and culture results

	Assays	Culture positive		Culture negative		Sensitivity	Specificity	PPV	NPV
		PCR+	PCR−	PCR+	PCR−				
MTB									
All	Genedia	23	7	1	319	76.7	99.7	95.8	97.9
	Anyplex	26	4	8	310	86.7	97.5	76.5	98.7
AFB smear positive	Genedia	16	2	1	1	88.9	50.0	94.1	33.3
	Anyplex	18	0	1	1	100.0	50.0	94.7	100.0
AFB smear negative	Genedia	6	5	0	288	54.5	100.0	100.0	98.3
	Anyplex	7	4	7	279	63.6	97.6	50.0	98.6
NTM									
All	Genedia	17	6	0	319	73.9	100.0	100.0	98.2
	Anyplex	17	6	2	310	73.9	99.4	89.5	98.1
AFB smear positive	Genedia	13	4	0	1	76.5	100.0	100.0	20.0
	Anyplex	14	3	0	1	82.4	100.0	100.0	25.0
AFB smear negative	Genedia	4	2	0	288	66.7	100.0	100.0	99.3
	Anyplex	3	3	2	279	50.0	99.3	60.0	98.9

Abbreviations: Anyplex, Anyplex plus MTB/NTM Detection kit; Genedia, Genedia MTB/NTM Detection kit; MTB, *Mycobacterium tuberculosis*; NPV, negative predictive value; NTM, nontuberculous mycobacteria; PCR, polymerase chain reaction; PPV, positive predictive value.

Of the 236 respiratory specimens cultured, 26 (11.0%) were positive for MTB and 22 (9.3%) were positive for NTM. Of the 137 non‐respiratory specimens cultured, 4 (2.9%) were positive for MTB and 1 (0.7%) for NTM. The sensitivity, specificity, positive predictive value (PPV), and negative predictive value (NPV) of each assay were calculated using the culture results as the “gold standard” (Table 1). For MTB detection, the sensitivity, specificity, PPV, and NPV of the Genedia kit were 76.7%, 99.7%, 95.8, and 97.9%, respectively, whereas for the Anyplex assay, these values were 86.7%, 97.5%, 76.5%, and 98.7%, respectively. For NTM detection, the sensitivity, specificity, PPV, and NPV of the Genedia assay were 73.9%, 100%, 100%, and 98.2%, respectively, and for the Anyplex assay, these values were 73.9%, 99.4%, 89.5%, and 98.1%, respectively.

In AFB smear‐positive specimens, the sensitivity, specificity, PPV, and NPV of the Genedia kit for the detection of MTB were 88.9%, 50.0%, 94.1%, and 33.3%, respectively, whereas those of Anyplex kit were 100.0%, 50.0%, 94.7%, and 100.0%, respectively. In AFB smear‐negative specimens, the sensitivity, specificity, PPV, and NPV of the Genedia kit for the detection of MTB were 54.5%, 100.0%, 100.0%, and 98.3%, respectively, whereas those of Anyplex kit were 63.6%, 97.6%, 50.0%, and 98.6%, respectively.

The degree of agreement between the two PCR assays in the detection of both MTB and NTM was measured by calculating the kappa (κ) coefficient. The value of kappa is 0.836, which is a statistically high agreement (Table 2).

**Table 2:** Correlations of two real‐time PCR kits

	Genedia			
	MTB	NTM	negative	total
Anyplex				
MTB	24	0	10	34
NTM	0	16	3	19
Negative	0	1	319	320
Total	24	17	332	373

Abbreviations: Anyplex, Anyplex plus MTB/NTM Detection kit; Genedia, Genedia MTB/NTM Detection kit; MTB, *Mycobacterium tuberculosis*; NTM, nontuberculous mycobacteria.

## 4 DISCUSSION

We evaluated the performance of the Genedia MTB/NTM Detection kit compared with the Anyplex plus MTB/NTM Detection kit. According to the results, sensitivity and specificity in MTB were 76.7% and 99.7% in Genedia, and 86.7% and 97.5 in Anyplex, respectively. Sensitivity and specificity in NTM were 73.9% and 100.0% in Genedia, and 73.9% and 99.4% in Anyplex, respectively. There were no statistically significant differences between the two tests.

A previous evaluation of the Genedia assay for MTB detection in respiratory samples reported sensitivity and specificity as 81.8% and 99.8%, respectively.^6^ Sensitivity and specificity of the Anyplex assay for detection of MTB in respiratory specimens have been reported as 87.5% and 98.2%.^7^ For both respiratory and non‐respiratory specimens, sensitivity and specificity were 71.0% and 94.9%.^8^ We found that sensitivity of both kits was lower with AFB smear‐negative samples, which is consistent with previous findings. Nine false‐positive results were detected in AFB smear‐negative specimens using the Anyplex assay, resulting in a lower PPV (76.5%) than that of the Genedia assay (95.8%). In another previous study, the TAT (Turn Around Time) of Genedia kit was about 140 minutes and showed high correlation and accuracy with conventional PCR and other real‐time PCR methods (AdvanSure kit, Real‐Q kit).^9^

In this study, the specificity and NPV calculated for AFB smear‐positive specimens were significantly lower than those for smear‐negative specimens. This may be due to the small number of specimens, which is a limitation of this study.

The United States Centers for Disease Control and Prevention (CDC) recommends that nucleic acid amplification testing (NAA), using a Food and Drug Administration (FDA) approved test, should be performed on at least one respiratory specimen from each patient with signs and symptoms of pulmonary TB.^3^ The Korean guidelines for TB recommend that when an individual is suspected of having pulmonary TB, NAA tests should be performed at least once in combination with AFB smear and culture.^4^ RT‐PCR kits using NAA techniques have been developed with increasing sensitivity and specificity than the initial product launch and have been used in many clinical laboratories with the advantage of obtaining rapid results.^5^

In summary, both the Genedia MTB/NTM Detection kit (Green Cross Medical Science) and the Anyplex plus MTB/NTM Detection kit (Seegene) have the capacity to report high sensitivity and specificity in a short time and both demonstrate a high concordance rate. We therefore propose that these kits can be used more widely in clinical laboratories. It is considered necessary to study the problem of increased false‐positive rate when targeting AFB‐negative samples. Further research is needed in near future, including identification of non‐detected and false‐positive samples in larger numbers of specimens.
